# Altered correlation of concurrently recorded EEG-fMRI connectomes in temporal lobe epilepsy

**DOI:** 10.1162/netn_a_00362

**Published:** 2024-07-01

**Authors:** Jonathan Wirsich, Giannina Rita Iannotti, Ben Ridley, Elhum A. Shamshiri, Laurent Sheybani, Frédéric Grouiller, Fabrice Bartolomei, Margitta Seeck, François Lazeyras, Jean-Philippe Ranjeva, Maxime Guye, Serge Vulliemoz

**Affiliations:** EEG and Epilepsy Unit, Division of Neurology, Geneva University Hospitals and University of Geneva, Geneva, Switzerland; Aix-Marseille Univ, CNRS, CRMBM 7339, Marseille, France; AP-HM CHU Timone, CEMEREM, Marseille, France; IRCCS Istituto delle Scienze Neurologiche di Bologna, Bologna, Italy; UCL Queen Square Institute of Neurology, Queen Square, London, UK; Swiss Center for Affective Sciences, University of Geneva, Geneva, Switzerland; Aix-Marseille Univ, INS, INSERM, UMR 1106, Marseille, France; AP-HM CHU Timone, Service d’épileptologie, Marseille, France; Department of Radiology and Medical Informatics, University of Geneva, Geneva, Switzerland

**Keywords:** Temporal lobe epilepsy, Concurrent EEG-fMRI, Resting state, Functional connectome, Multimodal data integration

## Abstract

Whole-brain functional connectivity networks (connectomes) have been characterized at different scales in humans using EEG and fMRI. Multimodal epileptic networks have also been investigated, but the relationship between EEG and fMRI defined networks on a whole-brain scale is unclear. A unified multimodal connectome description, mapping healthy and pathological networks would close this knowledge gap. Here, we characterize the spatial correlation between the EEG and fMRI connectomes in right and left temporal lobe epilepsy (rTLE/lTLE). From two centers, we acquired resting-state concurrent EEG-fMRI of 35 healthy controls and 34 TLE patients. EEG-fMRI data was projected into the Desikan brain atlas, and functional connectomes from both modalities were correlated. EEG and fMRI connectomes were moderately correlated. This correlation was increased in rTLE when compared to controls for EEG-delta/theta/alpha/beta. Conversely, multimodal correlation in lTLE was decreased in respect to controls for EEG-beta. While the alteration was global in rTLE, in lTLE it was locally linked to the default mode network. The increased multimodal correlation in rTLE and decreased correlation in lTLE suggests a modality-specific lateralized differential reorganization in TLE, which needs to be considered when comparing results from different modalities. Each modality provides distinct information, highlighting the benefit of multimodal assessment in epilepsy.

## Author Summary

The relationship between resting-state hemodynamic (fMRI) and electrophysiological (EEG) connectivity has been investigated in healthy subjects, but this relationship is unknown in patients with left and right temporal lobe epilepsies (l/rTLE). Does the magnitude of the relationship differ between healthy subjects and patients? What role does the laterality of the epileptic focus play? What are the spatial contributions to this relationship? Here we use concurrent EEG-fMRI recordings of 65 subjects from two centers (35 controls, 34 TLE patients), to assess the correlation between EEG and fMRI connectivity. For all datasets, frequency-specific changes in cross-modal correlation were seen in lTLE and rTLE. EEG and fMRI connectivities do not measure perfectly overlapping brain networks and provide distinct information on brain networks altered in TLE, highlighting the benefit of multimodal assessment to inform about normal and pathological brain function.

## INTRODUCTION

It now is consensus that multimodal integration of whole-brain imaging facilitates the clinical exploration of brain pathology. However, it is yet an open question how multimodal measures of pathological brain networks can help in epilepsy to guide clinical diagnosis, treatment, and brain surgery (Zijlmans et al., 2019). While epileptic phenomena are clinically characterized by altered brain rhythms and paroxysmal local discharges, recorded using the electroencephalogram (EEG), more widespread whole-brain functional alteration linked to epilepsy has been characterized by functional MRI (fMRI) (Centeno & Carmichael, 2014). In a clinical context the fast dynamics of EEG and the finer spatial resolution of fMRI can be used to investigate the hemodynamic changes correlated with epileptic spikes in order to obtain an improved spatial characterization of the epileptogenic network (Vulliemoz et al., 2009).

To investigate whole-brain functional network alterations associated with epilepsy, fMRI (Bettus et al., 2009; Ridley et al., 2015), EEG (Coito et al., 2015), and MEG (Li Hegner et al., 2018) have been successfully applied, but it remains unclear how results extracted from different modalities can be used together in a meta-analysis (Slinger et al., 2022; van Diessen et al., 2014). To translate basic research results derived from complex fMRI connectivity graph models into clinical management of patients with epilepsy, it is indispensable to better understand the correspondence between EEG and fMRI connectivity. Previous work suggests that connectivity in patients suffering from focal right and left temporal lobe epilepsies are differentially organized from a structural (Besson et al., 2014) and also from a functional point of view (EEG: Coito et al., 2015; fMRI: Ridley et al., 2015).

In healthy subjects, moderate correlations between concurrently recorded EEG and fMRI functional connectivity (FC_fMRI_ and FC_EEG_) exist (Deligianni et al., 2014; Wirsich et al., 2021), and EEG and fMRI connectivity dynamics are linked to each other (Wirsich et al., 2020b) while parts of the FC_EEG_ and FC_fMRI_ provide complimentary information (Wirsich et al., 2017, 2020a). The relationship between FC_fMRI_ and electrophysiological connectivity is not limited to FC_EEG_ but has been equally observed between FC_MEG_ and FC_fMRI_ (Brookes et al., 2011; Hipp & Siegel, 2015; for review, see Sadaghiani & Wirsich, 2020). Being able to extract both commonalties and discrepancies between FC_fMRI_ and FC_EEG_ is encouraging as they point in the direction that whole-brain networks extracted from clinical EEG can be generally used instead of a more expensive assessment with fMRI. As such, mapping FC_EEG_ and FC_fMRI_ into one graph space provides a framework to translate fMRI findings into the clinical setting of EEG recordings. For this purpose, it is necessary to understand if the relationship between EEG and fMRI is altered when comparing healthy subjects and patients with epilepsy. Alterations between healthy and pathological networks in electrophysiology and hemodynamics are complex, and specific alterations of the EEG-fMRI relationship have been reported in combination with several EEG frequency bands while the reproducibility of those individual studies remains unclear (Centeno & Carmichael, 2014). An unaltered FC_EEG_-FC_fMRI_ relationship would suggest that recording a single modality may be enough to characterize functional connectivity alterations in epilepsy, while a changed relationship would highlight the importance of multimodal exploration (Forsyth et al., 2019).

In this exploratory study, we sought to characterize the spatial correlation between whole-brain FC_EEG_ and FC_fMRI_ in order to understand if the cross-modal mapping of FC_EEG_ and FC_fMRI_ is modified in patients with epilepsy as compared to healthy controls. This will close the knowledge gap of how FC_fMRI_ and FC_EEG_ studies compare in focal epilepsies. The advantage of this approach is that the exact topology of reorganization is irrelevant: the spatial correlation of whole-brain EEG and fMRI connectivity will measure the topological alteration of networks that generalize across the patient group while omitting local patient-specific functional reorganization. We aimed to assess the reproducibility of our results by using two independently recorded EEG-fMRI datasets.

## METHODS

### Participants and EEG-fMRI Data Acquisition

We included patients with drug-resistant focal temporal lobe epilepsy with clear unilateral epileptic focus (clinically defined by combined information from imaging, interictal epileptiform discharges (IEDs), and seizure onset) alongside healthy controls with no history of neurological or psychiatric illness. To do so, we retrospectively used data from two independent centers using a 256-channel EEG setup in a 3T scanner (dataset will be referenced as 256Ch-3T) and a 64-channel EEG setup in a 3T scanner (dataset 64Ch-3T). We included data of resting-state concurrent EEG-fMRI acquisitions in a total of 35 healthy controls (64-3T: 14 and 256-3T: 21) and a total 34 patients diagnosed with drug-resistant epilepsy of the temporal lobe (TLE, 64-3T: *n* = 11 and 256-3T *n* = 23/distribution of left and right TLE: rTLE *n* = 18 and lTLE *n* = 16; for clinical information see Supporting Information Table S2).

**256Ch-3T**: 21 healthy subjects (7 females, mean age: 32, age range 24–47) with no history of neurological or psychiatric illness and 23 TLE patients (14 females, mean age: 34, age range 18–60, 13 lTLE and 10 rTLE) were recorded. Ethical approval was given by the local Research Ethics Committee (Commission Cantonale d’Ethique, Genève), and informed consent was obtained from all subjects. The control group has been previously analyzed in Wirsich et al. (2021).

A variable time period of resting-state simultaneous EEG-fMRI data were acquired for patient and control groups. In order to have a consistent recording length within the dataset we only analyzed the first 4 min and 58 s of each dataset (see Table S1; due to excessive muscle artifacts in the first 5 min of the recordings, one participant was analyzed in the period 5 min to 9 min and 58 s). Subjects were asked not to move, to remain awake, and keep their eyes closed during the resting-state scan. MRI was acquired using a 3 Tesla MR-scanner (Siemens Magnetom Trio/Siemens Magnetom Prisma, update of clinical scanner during protocol, see Table S1). The fMRI scan comprised the following parameters: GRE-EPI sequence, TR = 1,980/1,990/2,000 ms (for details see update of clinical scanner during protocol see Table S1), TE = 30 ms, 32 slices, voxel size 3 × 3 × 3.75 mm^3^, flip angle 90°. Additionally, an anatomical T1-weighted image was acquired (176 sagittal slices, 1.0 × 1.0 × 1.0 mm, TA = 7 min). EEG was acquired using a 258-channel MR-compatible amplifier (Electrical Geodesic Inc., Eugene, OR, USA, sampling rate 1 kHz), including 256 electrodes (Geodesic Sensor Net 256, referenced to Cz) and 2 ECG electrodes (bipolar montage, placed on the chest, crossing the heart). The scanner clock was time-locked with the amplifier clock (Mandelkow et al., 2006). An elastic bandage was pulled over the subjects’ heads and EEG caps to assure the contact of electrodes on the scalps. The MR-compatible amplifier was positioned to the left of the subject and EEG, and ECG cables were passed through the front end of the bore.

**64Ch-3T**: 14 healthy subjects (5 females, mean age: 31, age range 20–55) with no history of neurological or psychiatric illness and 11 TLE patients (6 females, mean age: 37, age range 22–54, 3 lTLE and 9 rTLE) were recorded. Ethical approval was given by local Research Ethics Committee (Comité de Protection des Personnes Marseille 2), and informed consent was obtained from all subjects. Data of the control group has been previously analyzed in Wirsich et al. (2017, 2020a).

In each subject, one run of 21-min resting-state simultaneous EEG-fMRI was acquired. We used the total length of the data for connectivity analysis. Subjects were asked not to move and to remain awake and keep their eyes closed during the resting-sate scan. MRI was acquired using a 3 Tesla MR-scanner (Siemens Magnetom Verio 3T). The fMRI scan comprised the following parameters: GRE-EPI sequence, TR = 3,600 ms, TE = 27 ms, 50 slices, voxel size 2 × 2 × 2.5 mm, flip angle 90°, total of 350 vols. Additionally, an anatomical T1-weighted image was acquired (208 sagittal slices, 1.0 × 1.0 × 1.0 mm, TA = 6 min 27 s).

EEG was acquired using a 64-channel MR-compatible amplifier (BrainAMP MR – Brain Products, Munich, Germany, sampling rate 5 kHz), 64 electrodes (referenced to FCz, 1 ECG electrode placed on the chest above the heart). The scanner clock was time-locked with the amplifier clock (Mandelkow et al., 2006). The amplifier was placed as far as possible behind the scanner, and the connector cables were fixed with sandbags to avoid distortions due to mechanical vibrations of the scanner.

### Data Processing

Data preprocessing was carried out as described in Wirsich et al. (2021). In order to assure comparability to this study, we applied the same EEG and fMRI connectivity measures.

#### Brain parcellation.

We used the Freesurfer toolbox (Fischl, 2012) to process the T1-weighted images (recon-all, v6.0.0 https://surfer.nmr.mgh.harvard.edu/) by performing nonuniformity and intensity correction, skull stripping, and gray/white matter segmentation. The cortex was parcellated into 68 cortical regions according to the Desikan(-Killiany) atlas (Desikan et al., 2006). Following the results of Farahibozorg et al. (2018), showing that the optimal size of parcellation to capture independent EEG signals contains around 70 regions, we decided to use the Desikan atlas as reference. While the 68 regions of the Desikan atlas do not take advantage of the high resolution of fMRI, we showed previously that EEG-fMRI correlations are less pronounced on with a more fine-grain Destrieux atlas (Wirsich et al., 2021).

#### fMRI processing.

Slice timing correction was applied to the fMRI time series. This was followed by spatial realignment both using the SPM12 toolbox (revision 7475; https://www.fil.ion.ucl.ac.uk/spm/software/spm12). The T1 images of each subject and the Desikan atlas were coregistered to the fMRI images (FSL-FLIRT 6.0.2, https://fsl.fmrib.ox.ac.uk/fsl/fslwiki [Jenkinson et al., 2012]). We extracted signals of no interest such as the average signals of cerebrospinal fluid (CSF) and white matter from manually defined regions of interest (ROI; 5-mm sphere, Marsbar Toolbox 0.44, https://marsbar.sourceforge.net) and regressed them out of the BOLD time series along with 6 rotation, translation motion parameters, and global gray matter signal (Wirsich et al., 2017). Then we band-pass filtered the time series at 0.009–0.08 Hz (Power et al., 2014). Like in Wirsich et al. (2021), we scrubbed the data using framewise displacement (threshold 0.5 mm, by excluding the superthreshold time frames) as defined by Power et al. (2012).

#### fMRI connectivity measures.

**Figure 1.**
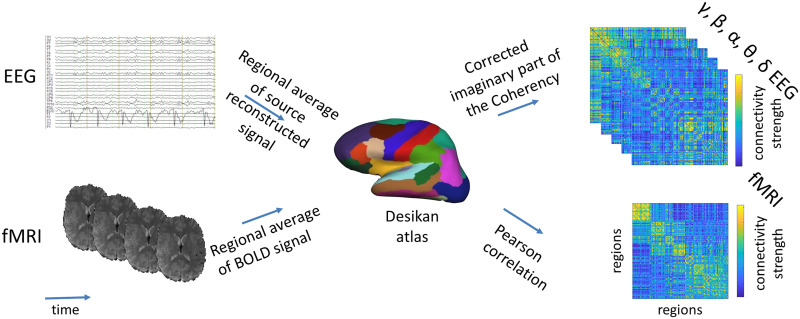
Overview on the construction of EEG and fMRI connectomes. EEG and fMRI data were parcellated into the 68 regions of the Desikan atlas (coregistered to each subject’s individual T1) as follows: for fMRI, the BOLD signal time course was averaged over the voxels in each region for each subject. The Pearson correlation of the region averaged fMRI-BOLD time course was calculated to build a function connectivity matrix/connectome (FC_fMRI_). For the EEG, the signal of each sensor was source reconstructed to the cortical surface (15,000 solution points) using the Tikhonov-regularized minimum norm. Then, the time courses of the solution points were averaged per cortical region. The corrected imaginary part of the coherency (ciCoh) of averaged EEG source signals were used to calculate FC_EEG_ for each subject (Figure adapted from Wirsich et al. (2021). Please refer to the Methods for a detailed description of each step).

Average time series of each region was then used to calculate FC_fMRI_ by taking the pair-wise Pearson correlation of each regions’ cleaned time course (see schema in Figure 1). The final connectivity matrix was constructed from the unthresholded values of the Pearson correlation.

#### EEG processing.

EEG data was preprocessed individually for the different setups.

**256Ch-3T**: EEG was corrected for the scanner gradient artifact by using template subtraction with optimal basis set and adaptive noise cancelation (Allen et al., 2000; Niazy et al., 2005), followed by pulse-related artifact template subtraction (Allen et al., 1998) using in-house code MATLAB code for ballistocardiogram peak detection as described in Iannotti et al. (2015). Electrodes placed on the cheeks and in the face were excluded from data analysis, resulting in a final set of 204 used electrodes. This was followed by manual ICA-based denoising (for manual removal of gradient and pulse artifact residuals, eye-blinks, muscle artifacts, infoMax, runICA-function EEGLab revision 1.29 [Bell & Sejnowski, 1995; Delorme & Makeig, 2004]).

**64Ch-3T**: The Brain Vision Analyzer 2 software (Brain Products, Gilching, Germany) was used for the following processing steps. EEG was corrected for the scanner gradient artifact using template subtraction, adaptive noise cancelation and downsampling to 250 Hz (Allen et al., 2000) followed by pulse-related artifact template subtraction (Allen et al., 1998). Then ICA-based denoising (for manual removal of gradient and pulse artifact residuals, eye-blinks and muscle artifacts, Fast ICA restricted mode with probabilistic sphering) was carried out. Data was segmented according to one TR of the fMRI acquisition (TR = 3,600 ms). The segments with obvious motion or residual pulse artifacts were semiautomatically excluded from further analysis (manually selected segments around automatically detected peak activity of min: <−300 *μ*V or max: >300 *μ*V [Wirsich et al., 2017]). Finally, the data was band-pass filtered with the signal at 0.3–70 Hz.

**Both datasets**: A trained neurologist (L.S.) visually inspected all EEG data to mark interictal epileptiform discharges (IEDs), IED segments were not removed but were used as a covariable in our analysis. Cleaned EEG data was imported and analyzed with Brainstorm software (Tadel et al., 2011), which is documented and freely available under the GNU general public license (https://neuroimage.usc.edu/brainstorm, version 15th January 2019).

**256Ch-3T**: (The following steps were already carried out in the Brain Vision Analyzer software for 64Ch-3T data.) Data was band-pass filtered at 0.3–70 Hz. Data was segmented according to one TR of the fMRI acquisition (TR = 1,980–2,000 ms; see Table S1). In order to minimize the effect of head motion, EEG epochs containing motion were automatically detected if the signal in any channel exceeded the mean channel time course by 4 standard deviations. Then the whole time course was visually inspected to exclude all segments clearly containing motion or residual pulse artifact from further analysis (Wirsich et al., 2021).

**Both datasets**: Channels that remained artifactual were removed from the analysis (without interpolation). Electrode positions and T1 were coregistered by manually aligning the electrode positions onto the electrode artifacts visible in the T1 image. A forward model of the skull was calculated based on the individual T1 image of each subject using the OpenMEEG BEM model (Gramfort et al., 2010; Kybic et al., 2005). Scalp, skull, and brain surfaces were included in the BEM model (1,922 vertices each) for the head model using conductivity values defined by default OpenMEEG parameters (scalp: 1, skull: 0.0125, brain: 1). The noise covariance was estimated by calculating the block-wise data covariance and averaging it over the whole recording. The EEG signal was re-referenced to the global average and projected into source space (15,000 solution points on the cortical surface) by using the Tikhonov-regularized minimum norm (Baillet et al., 2001) with the Tikhonov parameter set to 10% (Brainstorm 2018 implementation, with default parameters: assumed SNR ratio 3.0, using current density maps, constrained sources normal to cortex with signs flipped into one direction, depth weighting 0.5/max amount 10). Note that the Tikhonov parameter could be optimized (Hincapié et al., 2016), but in order to remain consistent with our previous study (Wirsich et al., 2021), we kept the default parameter. Finally, the source activity of each solution point was averaged in each cortical region of the Desikan atlas.

#### EEG connectivity measures.

For the duration of each segment (the duration of the respective TR of the dataset) the corrected imaginary part of the coherency (ciCoh [Ewald et al., 2012; Nolte et al., 2004; Pascual-Marqui, 2007; Pascual-Marqui et al., 2011]) of the source activity was calculated between each region pair (cortical regions only: Desikan atlas, 68 regions) using bins of 2-Hz frequency resolution (Wirsich et al., 2021) (Brainstorm implementation, version 15-01-2019; imaginary part was corrected by the real part of the coherence coh: *ciCoh* = Imcoh21−Recoh2 [Pascual-Marqui, 2007]; please note that this term has been originally named lagged coherence [Pascual-Marqui, 2007; Pascual-Marqui et al., 2011]). The significance of each coherence value was determined according to Thompson (1979): *p* = ${\left(1-coh\right)}^{\frac{dof-2}{2}}$. For each segment, connections with *p* > 0.05 were set to 0. The 2-Hz bins were averaged for five canonical frequency bands: delta (*δ* 0.3–4 Hz), theta (*θ* 4–8 Hz), alpha (*α* 8–12 Hz), beta (*β* 12–30 Hz), and gamma (*γ* 30–60 Hz). These frequencies were chosen as the FC_fMRI_-FC_M/EEG_ relationship is considered to be frequency specific (Colclough et al., 2016; Hipp & Siegel, 2015; Sadaghiani & Wirsich, 2020; Tewarie et al., 2016; Wirsich et al., 2021). The connectivity measure was chosen to match our previous studies in healthy subjects (Wirsich et al., 2017, 2020a, 2020b, 2021). We showed previously that similar results can be obtained using amplitude envelope correlations (Wirsich et al., 2021) according to (Brookes et al., 2011; Hipp & Siegel, 2015).

The connectivity values of each segment were then averaged across time for each participant into a single FC_EEG_ matrix (see schema Figure 1; while some connections were thresholded on the segment level no threshold was applied to the final matrix).

### Connectivity Analysis

#### Split-half and cross-dataset spatial correlation.

Spatial similarity of monomodal FC was assessed by correlating the split-half averages of the upper triangular of the connectivity matrix of each dataset and group. To do so, each participant was randomly assigned to two equally sized datasets and the correlation between the two split-averaged matrices was calculated for multiple iterations. We report the correlation averaged over each split-half iteration (5,000 iterations or in the case of group sizes *n* < 16 we calculated all possible combinations to split the dataset into two parts). As those split-half correlations depend on the group size, the results should be only used to qualitatively assess the data and compare them to the results of Wirsich et al. (2021), but not to assess differences between controls and patients. Monomodal cross-dataset spatial correlation was assessed by correlating group averages of each dataset with the respective participant group in the other dataset.

#### Network-based statistics of monomodal measures.

With the goal to better understand if the individual FC_fMRI_ and FC_EEG_ are altered across groups due to local and monomodal shifts of connectivity, we used a general linear model (GLM) and network-based statistics (NBS) (Zalesky et al., 2010) approach on each modality. For FC_EEG_ this was done for each frequency band. In detail, we built six GLMs with FC_fMRI_ (Fisher z-transformed), FC_EEG-*δ*_, FC_EEG-*θ*_, FC_EEG-*α*_, FC_EEG-*β*_, and FC_EEG-*γ*_ as response (dependent) variables, group label as regressor of interest (independent variable), and age, sex, and dataset site as regressors of noninterest. In detail, NBS was used to correct for multiple comparison errors that occur when running mass-univariate tests on each connection of the FC matrix (Desikan atlas *n* = 2,778 connections). The correction is carried out by defining an uncorrected first-level threshold and comparing the network size of the resulting network to the size of permuted networks derived from permuted group labels (this approach is equivalent to cluster-based correction in SPM; for more information, see Zalesky et al. [2010]). We tested for monomodal network changes between controls and patients by applying the following contrasts: controls > rTLE, controls > lTLE, controls < rTLE, and controls < lTLE (one-sided *t* test, connection first-level *t* score threshold T = 2, NBS-corrected threshold adapted to six models *p* < 0.05/6 ∼ 0.0083).

#### Cross-modal spatial EEG-fMRI connectivity correlation.

Cross-modal spatial correlations between FC_EEG_ and FC_fMRI_ of each group-averaged connectivity matrices were calculated. A group-averaged connectivity matrix was derived by averaging the pair-wise connectivity values of each individual in the group. To test if the cross-modal correlation of rTLE and lTLE patients was different to the one of healthy controls, we built a distribution of 5,000 averaged matrices by randomly switching the group labels (Wirsich et al., 2016). Previously, we demonstrated in healthy controls that the spatial relationship of EEG-fMRI connectivity can be robustly extracted when averaging around 7–12 subjects (Wirsich et al., 2021). This excellent reproducibility of averaged resting-state recordings was also recently demonstrated on large fMRI datasets (*n* > 1,000, *r* > 0.9 for average connectomes with *n* > 10; see supplementary figure 17 in Marek et al. [2022]). The number of lTLE patients in the 64Ch3T-dataset was only *n* = 3, and in consequence we did not carry out any group-averaged analysis using only subjects restricted this group/dataset combination.

To understand how the cross-modal correlation is influenced by age, sex, epilepsy duration (as epilepsy onset and duration are correlated, we decided to use only duration), etiology, and IEDs, we generated several bootstrapped distributions (with replacement, MATLAB bootstrap function, 1,000 iterations) of the average EEG-fMRI correlation. This bootstrapping method will generate an average value for each EEG and fMRI connection that can be used to generate a bootstrapped FC_EEG_-FC_fMRI_ correlation alongside subject-specific variables such as age (e.g., one bootstrap iteration might result in an EEG-fMRI correlation of *r* = 0.3 an average female/male ratio of 0.4 and an average age of 33.1, while the next iteration will end up with *r* = 0.35, ratio = 0.45 and average age of 34.2). Each iteration of the bootstrapped averages were then used in three linear models to identify the relationship of each bootstrapped averaged variable to the bootstrapped averaged EEG-fMRI correlation (Model I: controls-lTLE patients: r(EEG-fMRI) ∼ age + sex + group label + dataset site; Model II: controls-rTLE patients: r(EEG-fMRI) ∼ age + sex + group label + dataset site; Model III: patients: r(EEG-fMRI) ∼ age + sex + epilepsy duration + isHS + recorded IEDs per minute + group label + dataset site; isHS = binary dummy variable coding for hippocampal sclerosis or not; for etiology distribution other than HS, see patient description in Supporting Information Table S2; epilepsy duration is coded in full years, dataset site = dummy variable coding for 256Ch-3T or 64Ch-3T dataset). To test for significance of the contribution to the EEG-fMRI connectivity correlation, the T-value of each coefficient/variable in the linear model was compared to a null model that bootstrapped (1,000 iterations with replacement) the averages of the same model having the target variable permuted across the dataset (e.g., group labels switched between lTLE and controls, 5,000 iterations).

#### Spatial subnetwork contribution to the EEG-fMRI connectivity correlation.

To better understand the spatial contributions to cross-modal correlations of the whole brain, we split the FC-matrices into subnetworks of the seven intrinsic connectivity networks (ICNs) (visual, somato-motor, ventral attention, dorsal attention, fronto-parietal, limbic, and default mode) as defined by Yeo et al. (2011). For each subdivision, we individually assessed the cross-modal correlation of the intrasubnetwork connections in order to statistically compare the difference between controls and patients (lTLE < controls/rTLE > controls, permutation test of group labels, 5,000 iterations). Equally, we assessed the contribution of each connection to the total cross-modal correlation (Colclough et al., 2016; Wirsich et al., 2021). In brief, the relative spatial contribution *c* of each connection *i* is given by: *c*_*i*_ = $\frac{z_{i}^{x}z_{i}^{y}}{\sum_{i}z_{i}^{x}z_{i}^{y}}$ = $\frac{z_{i}^{x}z_{i}^{y}}{r}$ with $z_{i}^{x}=\frac{x_{i}-\langle x\rangle }{\sqrt{\sum_{i}{\left(x_{i}-\langle x\rangle \right)}^{2}}}$ and $z_{i}^{y}=\frac{y_{i}-\langle y\rangle }{\sqrt{\sum_{i}{\left(y_{i}-\langle y\rangle \right)}^{2}}}$ given the Pearson correlation coefficient of two vectors *x* and *y*: *r* = $\frac{\sum_{i}\left(x_{i}-\langle x\rangle \right)\left(y_{i}-\langle y\rangle \right)}{\sqrt{\sum_{i}{\left(x_{i}-\langle x\rangle \right)}^{2}}\sqrt{\sum_{i}{\left(y_{i}-\langle y\rangle \right)}^{2}}}$ = $\sum_{i}z_{i}^{x}z_{i}^{y}$. This spatial contribution was statistically compared between patients and controls (direction of the test was chosen according to the results of the FC_EEG_-FC_fMRI_ correlation lTLE < controls/rTLE > controls, permutation of group labels, 5,000 iterations).

#### Exploratory analysis of potential confounders.

In order to better understand the data, we added exploratory analysis of potential confounders. Previous studies have shown a strong relationship between connectivity strength and Euclidian distance of the regions (Ercsey-Ravasz et al., 2013; Roberts et al., 2016). In line with this, we have shown that Euclidian distance is a major predictor when comparing multimodal connectivity derived from EEG, fMRI, and diffusion MRI (Wirsich et al., 2017). We have previously shown that the correlation between Euclidian distance and FC_fMRI_ is increased in patients with rTLE as compared to controls (Wirsich et al., 2016).

In addition to using the IED rate in the bootstrapping model, we repeated the main analysis by excluding patients with a high IED rate (arbitrary defined by more than 1 IED/minute). In line with this, previous work has shown that patients with hippocampal sclerosis might be particularly likely to suffer from IEDs that are not detectable on the scalp (Bruzzone et al., 2022; Gavaret et al., 2004; Tao et al., 2007). As such, we ran again the main analysis for HS and non-HS patients only.

Correlation of FC_fMRI-_-FC_EEG_ could be modified by intrahemispheric connections specific to the ipsi- or contralateral side of the supposed epileptic focus (Bettus et al., 2011). We tested the impact of hemispheres by running the main analysis on each hemisphere separately. At the individual level, we demonstrated that individual FC_fMRI_-FC_EEG_ correlation is low (Wirsich et al., 2021); we tested if the differences observed for the average group connectome can be replicated on individual connectomes. All *p* values are reported as their uncorrected values, and the corresponding *p* value that passes Bonferroni correction threshold at *p* < 0.05 is explicitly stated alongside each individual analysis. Values that pass Bonferroni correction at *p* < 0.05 are considered to be significant.

## RESULTS

### Behavioral

No significant difference in head movement measured by framewise displacement (used for scrubbing cutoff at 0.5) (Power et al., 2012) was observed between rTLE versus controls and lTLE versus controls (two-sided *t* test, all *p* > 0.05 uncorrected, test carried out independently for each dataset). Equally, we did not observe any significant differences between rTLE versus controls and lTLE versus controls for the final number of scrubbed (deleted) fMRI volumes and EEG segments (two-sided *t* test, all *p* > 0.05 uncorrected). No participants were excluded for extensive movement. The average number scrubbed fMRI volumes was 1/150 volumes (range 0–10, data set 256Ch-3T) and 9/350 volumes (range 0–32, dataset 64Ch-3T). The average number of scrubbed EEG connectomes was 8/150 (range 0–41, dataset 256Ch-3T) and 23/350 (range 6–47, dataset 64Ch-3T).

### Monomodal Split-Half and Cross-Dataset Correlation

Intragroup monomodal consistency of the subject groups (split carried out separately for each dataset site and control, lTLE, and rTLE patient group) was accessed by randomly, splitting the dataset into two equally sized parts (5,000 iterations or all combinations in case the number of subjects in the group was *n* < 16) and spatially correlating the averaged FC_EEG_ and FC_fMRI_ matrices of each split. The correlation between FC_fMRI_ between each split half ranged from *r* = 0.88 (controls, dataset 64Ch3T) to *r* = 0.62 (rTLE patients, dataset 256Ch3T). The correlation between each FC_EEG_ split half ranged from *r* = 0.82 (FC_EEG-*β*_, controls, dataset 256Ch3T) to *r* = 0.28 (FC_EEG-*γ*_, rTLE patients, dataset 64Ch3T; for all results see Supporting Information Table S3).

### Monomodal Contributions

When using network-based statistics (Zalesky et al., 2010) to compare the monomodal whole-brain pair-wise connectivity of each individual, we could not find any significant differences between rTLE patients and healthy controls and lTLE patients and healthy controls. This was the case for both FC_fMRI_ as well as FC_EEG_ of all frequency bands (controls > rTLE, controls > lTLE, controls < rTLE and controls < lTLE, one-sided *t* test, connection level threshold T = 2, NBS-corrected threshold adapted to six models *p* < 0.05/6 ∼ 0.0083, the six models correspond to the monomodal test for FC_fMRI_ and FC_EEG_ of the five frequency bands).

### EEG-fMRI Correlation

**Figure 2.**
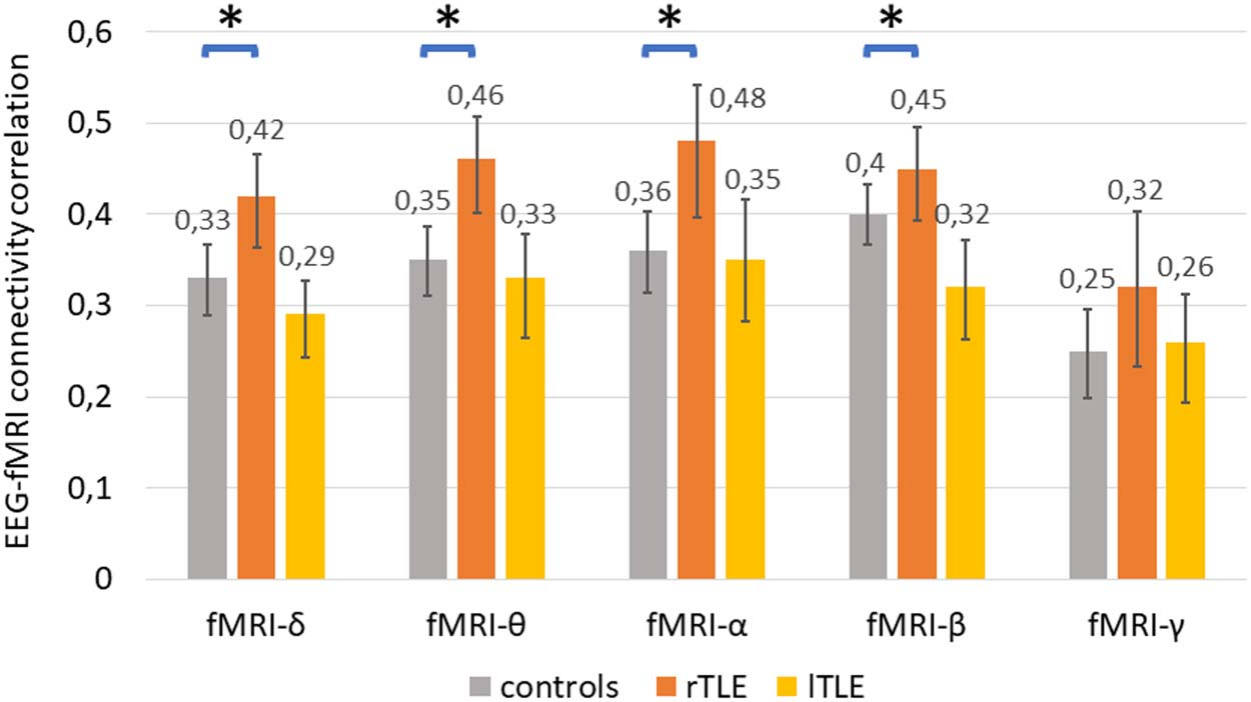
Cross-modal correlation between group-averaged FC_EEG_ and FC_fMRI_ (pooled across centers according to Wirsich et al. [2021]) using the Desikan atlas (*rTLE patients > controls Bonferroni threshold: *p* < 0.05/5 = 0.01, permutation test with 5,000 iterations; for all results, see Supporting Information Table S4. Black lines depict the 95% confidence interval of the bootstrapped mean FC_EEG_-FC_fMRI_ correlation with 10,000 iterations; for variability of correlation derived from permuted group labels, see Table S17).

In line with Wirsich et al. (2017, 2021), healthy controls moderately (*r* ∼ 0.3–0.4) correlated in the 256Ch-3T and 64Ch-3T dataset. EEG-fMRI correlation was also moderately correlated (*r* ∼ 0.3–0.4) in both patient groups (Figure 2 and Supporting Information Table S4).

**Figure 3.**
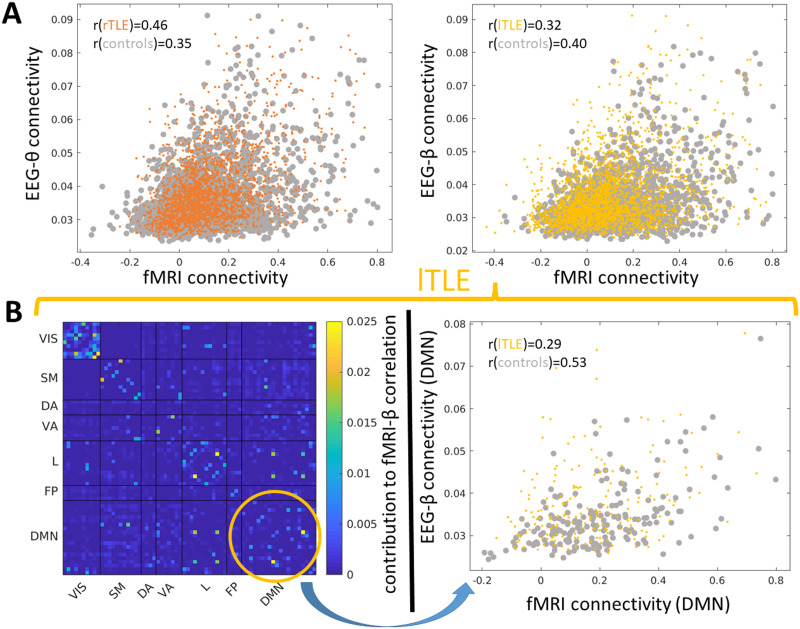
Scatter plots of all pair-wise FC_fMRI_ and FC_EEG_ connection strengths (each point samples the FC_EEG_ and FC_fMRI_ connection strength of one region pair of the group-averaged FC). (A, left) Significant FC_EEG_-FC_fMRI_ correlation differences in controls and rTLE patients in the *θ*-band (bootstrapped 95% confidence intervals with 10,000 iterations of mean FC_EEG_-FC_fMRI_ correlation controls: +0.036/−0.039 and rTLE: +0.047/−0.058) and (A, right) controls and lTLE patients in the *β*-band (bootstrapped 95% confidence intervals with 10,000 iterations of mean FC_EEG_-FC_fMRI_ correlation in controls: +0.032/−0.034 and lTLE; +0.051/−0.057). (B, left) Spatial contribution to FC_fMRI_-FC_EEG-*β*_ correlation of lTLE patients (yellow circle depicts the spatial contribution of the DMN network exhibiting a trend decrease in lTLE patients as compared to healthy controls *p* = 0.0074, uncorrected; Supporting Information Table S8 and Table S13). (B, right) Scatter plot of FC_fMRI_ and FC_EEG-*β*_ connection strengths in the DMN that are significantly less correlated in lTLE patients as compared to healthy controls (bootstrapped 95% confidence intervals with 10,000 iterations of controls: +0.056/−0.067 and lTLE: +0.104/−0.129); we did not find any significant local alterations of the cross-modal relationship when comparing rTLE patients to healthy controls (see Table S4); VIS: visual; SM: somato-motor; DA: dorsal attention; VA: ventral attention; L: limbic; FP: fronto-parietal; DMN: default mode network.

As compared to healthy controls, cross-modal correlation of rTLE patients was increased in FC_EEG-*δ*_, FC_EEG-*θ*_, FC_EEG-*α*_, and FC_EEG-*β*_ (corrected Bonferroni threshold: *p* < 0.05/5 = 0.01; see Figure 2 and Figure 3A). For lTLE patients, we observed no significantly altered correlation as compared to healthy controls (r(FC_fMRI_, FC_EEG_): lTLE < controls, corrected Bonferroni threshold: *p* > 0.05/5 = 0.01; Figure 3A).

When combining all subjects to a grand average (patient and control group), the multimodal correlation peaks at *r* ∼ 0.40 (except *γ*: *r* = 0.33; see first row of Table S4), as observed in Wirsich et al. (2021). As we have previously demonstrated, adding more healthy controls will generally increase the correlation (Wirsich et al., 2021); the higher correlation of rTLE (*n* = 17) compared to all pooled subjects (*n* = 35) makes the possibility that the result is driven by a random higher SNR of rTLE patients very unlikely (see Table S4). From a geometric point of view increased/decreased correlation between groups was generally accompanied by a trend of increased/decreased negative correlation with Euclidian distance of both modalities (Supporting Information Table S12; not the case for ED vs. FC_EEG-*β*_ and FC_EEG-*γ*_ correlation in rTLE vs. controls). Though the majority of patients did not show any IEDs (Table S9) in order to definitively exclude the effect of IEDs on this relationship, we showed that the results remain stable when excluding patients with more than 1 IED per minute (2 patients excluded; Table S10). When rerunning the analysis on patients with hippocampal sclerosis versus no hippocampal sclerosis, we observed that while results for rTLE patients were comparable, the results of lTLE group hint that HS patients drive the correlation of FC_fMRI_-FC_EEG-*β*_ (Table S14).

As predicted by the results of Wirsich et al. (2021), we did not observe any significant differences between groups when looking at individual FC_EEG_ and FC_fMRI_ instead of averaging (one-sided *t* test, Bonferroni corrected *p* > 0.05/5 = 0.01; the direction of the trends agrees with the group-averaged observations; see Table S16).

#### Bootstrapping the group-averaged FC_fMRI_-FC_EEG_ correlation.

Using bootstrapped group averages in a linear model in order to analyze how different resampling iterations with replacements change the average EEG-fMRI correlation, we observed that (1) in a model including controls and rTLE patients (controlling for age, sex, and dataset site): FC_fMRI_-FC_EEG-*δ*_/-FC_EEG-*θ*_/-FC_EEG-*α*_/-FC_EEG-*β*_ correlation was significantly increased for rTLE patients as compared to healthy controls (*p* < 0.05/5 = 0.01, Bonferroni corrected); (2) in a model including controls and lTLE patients (controlling for age, sex, and dataset site): FC_fMRI_-FC_EEG-*β*_ correlation was not significantly decreased (uncorrected trend only) for rTLE patients as compared to healthy controls (uncorrected *p* < 0.05); and (3) in a model including lTLE and rTLE patients (controlling for age, sex and dataset site, epilepsy duration, existence of hippocampal sclerosis and spikes/minute), we observed a significant increase of FC_fMRI_-FC_EEG-*δ*_/-FC_EEG-*θ*_/-FC_EEG-*α*_/-FC_EEG-*β*_ correlation when comparing rTLE to lTLE patients (rTLE > lTLE, *p* < 0.05/5 = 0.01, Bonferroni corrected). For detailed results of the bootstrap analysis, see Table S6.

#### Local spatial contributions to the FC_fMRI_-FC_EEG_ correlation.

We then compared the FC_fMRI_-FC_EEG_ correlation of subnetworks that take only into account connections from one specific ICN between TLE patients and healthy controls. We observed that when comparing lTLE patients to healthy controls, the FC_fMRI_-FC_EEG-*β*_ correlation was significantly decreased for lTLE patients in the default mode network (DMN) (lTLE < controls, permutation of group labels, 5,000 iterations, *p* < 0.05/(5 * 7) = 0.0014, corresponding to a Bonferroni threshold *p* < 0.05; Figure 3B and Table S8), no significant alterations were observed comparing rTLE patients to healthy controls (rTLE > controls, permutation of group labels, 5,000 iterations, *p* > 0.05/(5 * 7) = 0.0014, corresponding to a Bonferroni threshold *p* > 0.05).

When comparing the spatial contribution to the global EEG-fMRI connectome correlation (group averaged) between lTLE patients and healthy controls, we observed no significantly decreased spatial contribution (see Figure 3B and Table S8) (lTLE < controls, permutation of group labels, 5,000 iterations, *p* > 0.05/(5 * 7) = 0.0014, corresponding to a Bonferroni threshold of *p* < 0.05). When comparing rTLE patients with healthy controls, we observed no shift in contribution to the global correlation (permutation of group labels, 5,000 iterations, *p* > 0.05/(5 * 7) = 0.0014, corresponding to a Bonferroni threshold of *p* > 0.05). Post hoc testing controlling for clinical and demographic parameters and center on FC_EEG-*β*_FC_fMRI_ in lTLE versus healthy controls showed that both spatial correlation and spatial contribution are significantly decreased (*p* < 0.05; Table S13). When restricting the analysis to the intrahemispheric contributions of each hemisphere only we observed results comparable to the whole-brain analysis (Table S15).

## DISCUSSION

This study, based on simultaneously recorded EEG and fMRI functional connectivity in patients with temporal lobe epilepsy and healthy controls in two independent datasets, characterized how a whole-brain network approach in epilepsy relates between both modalities. We replicated the moderate relationship between whole-brain FC_fMRI_ and FC_EEG_ in healthy controls (Wirsich et al., 2021) and we confirmed for the first time that this relationship also exists in patients with epilepsy. While networks of rTLE patients show a widespread change of the relationship across EEG frequency bands, the networks of lTLE patients have a global relationship of EEG and fMRI connectivity more similar to controls. Nevertheless, alterations between lTLE patients and healthy controls were observed locally (in particular the DMN) and were linked to FC_EEG-*β*_. This suggests that functional network reorganization across multiple timescales undergoes a more widespread or heterogeneous change in rTLE patients, impacting the relationship between EEG and fMRI, while alterations of the multimodal relationship are more homogenously localized in lTLE patients.

### Monomodal Relationship

For both datasets, and in line with our previous research (Wirsich et al., 2021), we showed that monomodal intragroup correlation was high (FC_fMRI_) to moderate (FC_EEG-*γ*_). When comparing connection-wise differences in networks between patients and controls, we were unable to observe any significant differences between controls versus lTLE patients and controls versus rTLE patients. This is opposed to our previous findings in rTLE (Wirsich et al., 2016), where we observed FC_fMRI_ differences in rTLE patients versus controls (though using a high-resolution 512 region atlas as opposed to the low-resolution atlas of 68 regions used in this study). The difficulty of identifying a consistent localized network across patients reflects the general heterogeneity of network neuroscience literature in epilepsy, which can be very sensitive to individual methodological choices analyzing resting-state connectivity (Centeno & Carmichael, 2014; Royer et al., 2022; Slinger et al., 2022; van Diessen et al., 2014). In summary, we observed that spatial localization of monomodal FC differences lack a spatial homogeneity that can be detected with the small group size of 34 patients used here. The heterogeneity of connection-wise alterations of individual connections observed here can potentially be mediated by describing the network topologically using graph theoretic descriptions (Carboni et al., 2020; Ridley et al., 2015; Wirsich et al., 2016, 2021).

### FC_fMRI_-FC_EEG_ Correlation

The simplest way to compare brain networks derived from different modalities on a topological level is by using the spatial correlation of the connectivity (Honey et al., 2009; Wirsich et al., 2017). We observed significantly increased global FC_fMRI_-FC_EEG_ correlation in rTLE as compared to healthy controls. Conversely, global alterations between lTLE patients and controls were restricted in timescale to the FC_fMRI_-FC_EEG-*β*_ relationship, which was found to be locally dominant in the DMN. This is in line with the observation of Coito et al. (2015), Zhao et al. (2022), and Lee et al. (2018) showing that FC_EEG_ of rTLE patients undergoes more widespread alterations in brain networks than those of lTLE patients.

This finding of functional alterations affecting regions remote to the epileptic focus, resulting in global shift of functional networks altered by epilepsy as a function of the laterality of the epilepsy, is further supported by a recent multicentric study showing that, while atrophy in lTLE is more restrained to the ipsilateral side while in rTLE, both the ipsi and contralateral sides are affected (Park et al., 2022). From a structural point of view, we previously observed that FC_fMRI_ of rTLE patients is more closely related to structural connectivity derived from diffusion MRI than healthy controls (Wirsich et al., 2016). Here we replicated our previous findings (Wirsich et al., 2016) in two new datasets, showing that the Euclidian distance – FC_fMRI_ correlation is indeed significantly increased for rTLE patients versus healthy controls. This result was extended by demonstrating that the FC-ED correlation parallels a trend in the same direction in both modalities, when compared to the FC_EEG_-FC_fMRI_ correlation (e.g., decrease of the FC_EEG-*β*_-ED and FC_fMRI_), absolute correlation is paralleling with a decrease of FC_fMRI_-FC_EEG-*β*_ correlation in lTLE patients versus healthy controls (Table S12).

Together with the results in the current study in rTLE patients, this points to a general increase of correlation between both structural and functional connectivity across different temporal scales. Interestingly, for the FC_EEG_-FC_fMRI_ correlation, this does not seem to be the case in lTLE patients. As the results seem to be linked to the geometrical constraints (close connections having strong connectivity) imposed by the ED of regions (Ercsey-Ravasz et al., 2013; Roberts et al., 2016), future work should validate if this is also true for the structure-function relationship.

We previously demonstrated that FC_fMRI_ and FC_EEG_ hold both distinct and mutual information (Wirsich et al., 2017, 2020a). Though we observed a moderate correlation between FC_fMRI_ and FC_EEG_ for both healthy controls and TLE patients, this work confirms that FC_EEG_ and FC_fMRI_ studies do not measure exactly the same properties in line with the disconnect between FC_fMRI_ and FC_EEG_ graph analysis literature (Slinger et al., 2022). The results of our study stress that the relationship between FC_fMRI_ and FC_EEG_ is only partial and, more importantly, alters with the lateralization of epilepsy, limiting the direct comparability of EEG and fMRI connectome studies.

### Spatial Contribution of FC_fMRI_-FC_EEG_ Correlation

From a structural parcellation point of view, asymmetries between left and right temporal lobe have been widely described (Van Essen et al., 2012). Rather than a limitation of the functional repertoire (Wirsich et al., 2016), the differential spatial contributions in rTLE and lTLE patients suggest different adaptations of normal healthy functional networks to epilepsy, for example, more healthy bilateral functional integration of right temporal lobe versus a more localized function of the left temporal lobe (Raemaekers et al., 2018). Looking exclusively at the DMN, Haneef et al. (2012) observed that local changes of fMRI connectivity are larger in lTLE as compared to rTLE. In line, we observed that the decrease in EEG-fMRI connectivity relationship was linked locally to the DMN in lTLE but not in rTLE. We extend the observation of Haneef et al. (2012) by also showing that the multimodal connectivity reorganization is linked to a local change of FC_fMRI_-FC_EEG-*β*_ correlation in lTLE. While the results of lTLE versus controls suggest a link to DMN and FC_fMRI_-FC_EEG-*β*_ alterations, the rTLE versus controls showed no clear pattern of spatial or spectral specificity.

The cognitive consequences of differential reorganization in rTLE versus lTLE are, for example, illustrated by the results of Drane et al. (2013), demonstrating that while rTLE patients have problems with recognizing famous faces, lTLE patients rather have problems naming them. From a physiological point of view, the results are also in line with the general asymmetry of connectivity in temporal regions, resulting in increased local connectivity in the left hemisphere when compared to the right hemisphere (Raemaekers et al., 2018).

### Implications for Clinical Research

While we looked only at temporal lobe epilepsy, the observed lateralized discrepancy in the relationship of FC_EEG_ and FC_fMRI_ might not be limited to TLE but could apply more generally to the lateralization of the epileptic zone in epilepsy (Ridley et al., 2015). Better understanding of markers of laterization is needed, as it has been proposed that the degree of lateralization is linked to the outcome of epilepsy surgery (Negishi et al., 2011). Furthermore, this feature might not only be a sensitive marker restricted to epilepsy, but it might be also linked to lateralization of brain dysfunction (e.g., one could see the same effect in lateralized tumors or strokes that alter the brain network). Further studies would be needed to better understand the relationship in EEG and fMRI in other focal neuropathologies.

Using a bootstrapping approach, we did not observe that IED rate and epilepsy duration contribute significantly to the alteration of the EEG-fMRI relationship, suggesting that those parameters do modulate FC_fMRI_ and FC_EEG_ in a similar way between differently lateralized epilepsies. For IED contributions, this was to be expected from previous studies showing limited to no effect on connectivity when removing IED-containing epochs (Bartolomei et al., 2013; Bettus et al., 2008; Iannotti et al., 2016). We note that our supplementary post hoc analysis provides some evidence that patients with hippocampal sclerosis versus no HS potentially also modifies the multimodal correlation in lTLE patients. This exploratory finding should be validated in a future study. Consequently, the relationship of FC_fMRI_ and FC_EEG_ might provide a potential additional clinical marker to determine lateralization (Douw et al., 2019; Sadaghiani & Wirsich, 2020). Our results are encouraging as they generalize across two datasets, and future work should validate if the EEG-fMRI can be clinically used to determine if a patient has a lateralization of epilepsy in the left or right hemisphere.

### Methodological Considerations

From a spatial point of view, reconstructing EEG brain activity from deep cortical regions (such as the hippocampus) is still a subject of discussion (Pizzo et al., 2019). As such, our approach to symmetrically integrate FC_fMRI_ and FC_EEG_ was limited to the temporal lobe without the hippocampus as defined by Desikan et al. (2006) and Yeo et al. (2011) but including neighboring cortical regions such as the parahippocampal gyrus and temporal pole. As improving SNR of FC_EEG_ from hippocampal regions is still ongoing research, future work might profit from integrating monomodal FC_fMRI_ asymmetrically in this framework.

It has been previously shown that sleep can modify fMRI connectivity (Kaufmann et al., 2006; Tagliazucchi & van Someren, 2017; Wirsich et al., 2018). Tagliazucchi and Laufs (2014) showed that sleep is likely to occur in some participants as soon as 5 minutes into the scan. We previously observed that the EEG-fMRI relationship is not altered by cutting down longer sessions to 5 minutes (Wirsich et al., 2021). Therefore, we assumed that potential sleep or vigilance variation do not confound our analysis, but it might be worth checking this in future studies.

Furthermore, while separating rTLE patients between the two recording sites, we demonstrated that results exist individually for each site. We included only three lTLE patients for the 64Ch-3T dataset; nevertheless, using the proposed bootstrap approach, we did not observe any systematic effects of dataset site when pooling all the subjects together. On the other side, the boostrapping approach showed that the FC_fMRI_-FC_EEG_ relationship is also influenced to a lesser extent by sex. A larger healthy control cohort would be needed to systematically analyze this effect.

In this study we selected only clear cases of lateral temporal lobe epilepsy sampled out of a database of ∼200 EEG-fMRI recordings for the 256Ch3T dataset and ∼60 recordings for the 64Ch3T dataset to assure relative homogeneity of the groups. The final group of 34 patients was the most homogenous group with a reasonable sample size. However, when comparing TLE patients to controls, we demonstrated global changes of the FC_fMRI_ and FC_EEG_ relationship, and we were unable to extract a common network of reorganization based on pair-wise connections (both for EEG and fMRI). Better understanding of individual functional networks linked to epilepsy beyond the group-averaged approach taken here (Marek et al., 2022; Wirsich et al., 2016, 2021) will need a larger database with data pooling in an even more multicentric approach (Marek et al., 2022; Slinger et al., 2022). While the approach is common to track connectome relationships (Goñi et al., 2014; Honey et al., 2009; Wirsich et al., 2021), Marek et al. (2022) even encourage averaging of at least 10 subjects to reliably track functional connectivity of smaller samples (< 1,000 participants). Averaging is further motivated by a meta-analytical approach by Crossley et al. (2014) that also suggests common brain regions altered in TLE versus controls.

A larger multicentric approach would equally help to characterize the effect of individual antiseizure medication treatment on FC (Wandschneider & Koepp, 2016; Xiao et al., 2019), which was not taken into account. This effect is potentially negligible in our data as both rTLE and lTLE patients will undergo the comparable treatment, and previously measured drug effects on the EEG-fMRI correlation were observed to be small (Forsyth et al., 2019). However, a systematic characterization of a medication effect (Wirsich et al., 2018) is still missing in the research field of characterizing functional networks in focal epilepsies.

### Conclusions

In this study we investigated the FC_fMRI_-FC_EEG_ correlation in healthy controls and in TLE patients. We observed that monomodal alterations between controls and TLE are hard to track. However, when looking at the spatial correlation between FC_fMRI_ and FC_EEG_, we were able to demonstrate global alterations between rTLE patients and healthy controls, while alterations between lTLE patients and controls were more local. This demonstrates the differential organization of mono-lateral focal epilepsy of the same type that needs to be considered when comparing EEG to fMRI connectivity. It also demonstrates that each modality provides distinct information, highlighting the benefit of multimodal assessment in epilepsy. This property of distinct topological patterns depending on the lateralization of the epilepsy could be taken into account when clinically defining the epileptic focus of patients.

## ACKNOWLEDGMENTS

We would like to thank the reviewers for their kind comments motivating additional exploratory post hoc analyses that provide further insight into the data (section Exploratory analysis of potential confounders). Additional funding was provided by the Fondation Leenaards (https://dx.doi.org/10.13039/501100006387), the Louis-Jeantet Foundation (https://dx.doi.org/10.13039/501100001706), and the Centre d’Imagerie BioMédicale (CIBM) of the UNIL, UNIGE, HUG, CHUV, and EPFL.

## SUPPORTING INFORMATION

Supporting information for this article is available at https://doi.org/10.1162/netn_a_00362.

## AUTHOR CONTRIBUTIONS

Jonathan Wirsich: Conceptualization; Data curation; Formal analysis; Funding acquisition; Investigation; Methodology; Project administration; Resources; Software; Validation; Visualization; Writing – original draft; Writing – review & editing. Giannina Rita Iannotti: Data curation; Resources; Software; Writing – review & editing. Ben Ridley: Data curation; Writing – review & editing. Elhum Shamshiri: Data curation. Laurent Sheybani: Data curation; Writing – review & editing. Frédéric Grouiller: Data curation; Software; Writing – review & editing. Fabrice Bartolomei: Data curation; Resources. Margitta Seeck: Data curation; Resources. François Lazeyras: Data curation; Resources. Jean-Philippe Ranjeva: Data curation; Resources. Maxime Guye: Data curation; Resources. Serge Vulliemoz: Data curation; Funding acquisition; Resources; Supervision; Validation; Writing – review & editing.

## FUNDING INFORMATION

Serge Vulliemoz, Schweizerischer Nationalfonds zur Förderung der Wissenschaftlichen Forschung (https://dx.doi.org/10.13039/501100001711), Award ID: CRSII5_209470. Serge Vulliemoz, Schweizerischer Nationalfonds zur Förderung der Wissenschaftlichen Forschung (https://dx.doi.org/10.13039/501100001711), Award ID: 192749. Margitta Seeck, Schweizerischer Nationalfonds zur Förderung der Wissenschaftlichen Forschung (https://dx.doi.org/10.13039/501100001711), Award ID: 163398. Margitta Seeck, Schweizerischer Nationalfonds zur Förderung der Wissenschaftlichen Forschung (https://dx.doi.org/10.13039/501100001711), Award ID: 180365. Frederic Grouiller, Schweizerischer Nationalfonds zur Förderung der Wissenschaftlichen Forschung (https://dx.doi.org/10.13039/501100001711), Award ID: 188769. Laurent Sheybani, Schweizerischer Nationalfonds zur Förderung der Wissenschaftlichen Forschung (https://dx.doi.org/10.13039/501100001711), Award ID: P500PM_206720. Jonathan Wirsich, Faculté de Médecine, Université de Genève (https://dx.doi.org/10.13039/501100020971), Award ID: MA2020. Maxime Guye, Agence Nationale de la Recherche (https://dx.doi.org/10.13039/501100001665), Award ID: CONNECTEPI.

## DATA AVAILABILITY STATEMENT

All connectomes are available on Zenodo (https://doi.org/10.5281/zenodo.10470710 [Wirsich et al., 2022]). Raw data can be made fully available on request within the limits of ethical regulations of Switzerland and France. As such the project of the requesting party will need to undergo a formal ethical approval procedure (on request to SV and 256Ch-3T dataset or to MG for the 64Ch-3T dataset). Code is available at Github (https://github.com/jwirsich/eeg-fmri-tle).

## Supplementary Material



## TECHNICAL TERMS

Functional connectivity (FC): Denoting the temporal dependency of signal time courses at the systems level d (e.g., from fMRI, EEG, or MEG) measured from distributed brain regions.
Connectome: A whole-brain map of structural or functional neural connectivity. At the systems level, connections are typically established among brain regions, e.g., of a brain atlas.
Intrinsic connectivity networks (ICNs): Networks that spontaneously exhibit temporal dependency among neural activity time courses of their distributed regions. Regions of a given ICN also coactivate in response to the same cognitive demands.
Default mode network (DMN): A network or ICN of distributed brain areas that show increased activation as external cognitive demands diminish.
