# Supplementary material for: Altered correlation of concurrently recorded EEG-fMRI connectomes in temporal lobe epilepsy
Source: Netw Neurosci. 2024 Jul 1;8(2):466–85. doi: 10.1162/netn_a_00362 (PMC11142634; doi:10.1162/netn_a_00362)
Supplement: Supplementary file 1 [file netn-8-2-466-s001.pdf]

Supplementary Information: Altered correlation of concurrently recorded EEG-fMRI connectomes in temporal lobe epilepsy

Jonathan Wirsich[1], Giannina Rita Iannotti[1], Ben Ridley[2,3,4], Elhum A Shamshiri[1], Laurent Sheybani[1,5], Frédéric Grouiller[6], Fabrice Bartolomei[7,8], Margitta Seeck[1], François Lazeyras[9], Jean-Philippe Ranjeva[2,3], Maxime Guye[2,3], Serge Vulliemoz[1]

[1]EEG and Epilepsy Unit, University Hospitals and Faculty of Medicine of Geneva, Geneva, Switzerland

[2]Aix-Marseille Univ, CNRS, CRMBM 7339, Marseille, France

[3]AP-HM CHU Timone, CEMEREM, Marseille, France

[4]IRCCS Istituto delle Scienze Neurologiche di Bologna, Bologna, Italy

[5]UCL Queen Square Institute of Neurology, Queen Square, London, UK

[6]Swiss Center for Affective Sciences, University of Geneva, Geneva, Switzerland

[7]Aix-Marseille Univ, INS, INSERM, UMR 1106, Marseille, France

[8]AP-HM CHU Timone, Service d'épileptologie, Marseille, France

[9]Department of Radiology and Medical Informatics, University of Geneva, Geneva, Switzerland

Dataset	Scanner	TR	Resolution	Number of controls	Number of patients
**256Ch-3T**	Siemens Trio	1980ms	3x3x3.75mm	0	8
**256Ch-3T**	Siemens Trio	1990ms	3x3x3.75mm	17	10
**256Ch-3T**	Siemens Trio	2000ms	3x3x3.75mm	0	1
**256Ch-3T**	Siemens Prisma	1990ms	3x3x3.75mm	4	4
**64Ch-3T**	Siemens Magnetom Verio	3600ms	2.0x2.0x2.5mm	14	11

SI Table 1: Scanner setup for both centers. The scanner of the 256Ch-3T dataset received a scanner update during acquisition (data from years 2010-2019), which resulted in a slight change of acquisition parameters

#patient	Epilepsy lateralization	sex	age	Epilepsy onset (years)	Epilepsy duration (years)	Imaging
**256Ch-3T**						
1	left	M	18	8	10	Asymmetry of the temporal lobe
2	left	F	40	15	25	HS
3	left	M	32	23	9	Incomplete rotation of hippocampus
4	left	F	61	16	54	HS
5	left	M	44	17	27	HS
6	left	M	27	23	4	MR normal
7	left	F	26	13	13	HS
8	left	F	43	12	31	HS
9	left	M	26	18	8	HS
10	left	F	36	28	8	Tumor
11	left	F	42	17	25	HS
12	left	M	30	22	8	MR normal
13	left	F	29	16	13	MR normal
14	right	M	24	15	9	Vascular
15	right	F	32	9	23	HS
16	right	M	37	20	17	HS
17	right	F	29	27	3	HS
18	right	F	52	12	40	HS
19	right	F	30	25	5	CD
20	right	F	22	17	5	HS

21	right	F	60	25	35	HS
22	right	M	27	23	4	MR normal
23	right	M	36	5	32	HS
**64Ch-3T**						
24	left	M	22	19	3	MR normal
25	left	F	44	28	16	HS
26	left	M	54	54	1	MR normal
27	right	M	36	6	30	MR normal
28	right	F	27	11	17	Hypersignal amygdala
29	right	F	50	23	37	MR normal
30	right	M	31	29	2	Hypertrophy amygdala
31	right	M	35	12	32	HS
32	right	F	21	9	12	HS
33	right	F	38	12	26	Atrophy
34	right	F	49	20	29	Cavernoma

SI Table 2: Patient description HS: hippocampal sclerosis, CD: cortical dysplasia

	**fMRI**	**delta**	**theta**	**alpha**	**beta**	**gamma**
**Controls**						
Split half 256Ch3T	0.87	0.74	0.78	0.75	0.82	0.49
Split half 64Ch3T	0.88	0.81	0.71	0.56	0.58	0.35
Cross dataset	0.77	0.73	0.67	0.64	0.68	0.44
**lTLE**						
Split half 256Ch3T	0.79	0.72	0.74	0.76	0.82	0.59
**rTLE**						
Split half 256Ch3T	0.62	0.68	0.76	0.76	0.80	0.56
Split half 64Ch3T	0.78	0.59	0.47	0.38	0.49	0.28
Cross dataset	0.67	0.66	0.58	0.45	0.60	0.25

SI Table 3: Splithalf and crossdataset spatial correlation of monomodal FC_{EEG} and FC_{fMRI} (in the case of split-half correlation: random permutations of splithalf assignments with 5000 iterations, in case of n<16 all possible combinations to split the group into two halves). The crossdataset correlation is defined as the correlation of the group-averaged connectivity matrix of each dataset.

	delta	theta	alpha	beta	gamma
**All**	0.37	0.40	0.42	0.42	0.31
**Controls**	0.33	0.35	0.36	0.40	0.25
**lTLE**	0.29	0.33	0.35	0.32	0.26
**rTLE**	0.42	0.46	0.48	0.45	0.32
**P (5000 iterations)**	**delta**	**theta**	**alpha**	**beta**	**gamma**
**Controls > lTLE**	0.26	0.49	0.70	**0.039**	0.8568
**Controls < rTLE**	**0.001**	**<0.0002**	**<0.0002**	**0.0048**	0.015

SI Table 4: Spatial correlation between FC_{fMRI} and FC_{EEG} for each frequency band averaged for each group across both datasets (5000 permutations of group labels, significant results marked in dark green bold: Bonferroni threshold p<0.05/5=0.01), Light green: Uncorrected threshold p<0.05).

	delta	theta	alpha	beta	gamma
**256Ch-3T**					
Controls	0.31	0.33	0.35	0.37	0.23
lTLE	0.28	0.32	0.33	0.31	0.23
rTLE	0.38	0.39	0.41	0.39	0.31
**64Ch-3T**					
Controls	0.29	0.27	0.22	0.29	0.14
rTLE	0.36	0.38	0.31	0.33	0.15
**P (5000 iterations)**	**delta**	**theta**	**alpha**	**beta**	**gamma**
**256Ch-3T**					
Controls > lTLE	0.40	0.56	0.59	0.058	0.65
Controls < rTLE	**0.0072**	0.015	0.017	0.074	**0.0084**
**64Ch-3T**					
Controls < rTLE	0.017	0.016	0.079	0.0826	0.034

SI Table 5: Spatial correlation between FC_{fMRI} and FC_{EEG} for each frequency band for each dataset (rows for lTLE/64Ch-3T are not reported as only group is consisting of 3 patients, 5000 permutations of group labels, significant results marked in dark green bold: Bonferroni threshold p<0.05/5=0.01), Light green: Uncorrected threshold p<0.05.

	**delta**	**theta**	**alpha**	**beta**	**gamma**
**Controls & lTLE**					
Age	0.4508	0.2060	0.1070	0.2630	0.3136
Sex	0.0032	0.0214	0.0114	0.0620	0.1282
Controls vs. lTLE	0.4030	0.2744	0.1456	0.02316	0.1466
256Ch-3T vs. 64Ch-3T	0.2236	0.3550	0.0132	0.4398	0.1466
**Controls & rTLE**					
Age	0.1434	0.1522	0.3	0.0844	0.3588
Sex	0.0018	0.0066	0.2658	0.0412	0.1536
Controls vs. rTLE	***0.0006***	***<0.0002***	***0.0002***	**0.0018**	0.025
256Ch-3T vs. 64Ch-3T	0.2748	0.4356	0.028	0.386	0.0078
**lTLE & rTLE**					
Age	0.3746	0.1522	0.065	0.2442	0.2928
Sex	0.4386	0.2542	0.1068	0.4464	0.055
HS vs. Non-HS	0.257	0.0138	0.0074	0.0142	0.0112
Epilepsy Duration	0.2206	0.3074	0.4966	0.2398	0.2492
IED/Min	0.2726	0.0778	0.2204	0.1398	0.2018
lTLE vs. rTLE	***0.0002***	**0.0008**	**0.0032**	***0.0006***	0.0188
256Ch-3T vs. 64Ch-3T	0.2148	0.4544	0.0684	0.352	0.005

SI Table 6: P-Values of permutation test with 5000 iterations of linear model coefficients when bootstrapping averages on contrast of interest controls vs. lTLE/rTLE (dark green Bold: Bonferroni threshold p<0.05/5=0.01, Light green: Uncorrected threshold p<0.05, contrast of interest marked in light gray) and lTLE vs. rTLE (dark green Bold: Bonferroni threshold p<0.05/5=0.01, Light green: Uncorrected threshold p<0.05, only for contrast of interest marked in light gray). The Permutation test is carried out by switching labels of interest. Significant values are marked in BOLD italics. HS: hippocampal sclerosis

**lTLE<Controls**	**delta**	**theta**	**alpha**	**beta**	**gamma**
VIS	0.4080	0.6452	*0.0990*	0.0494	0.0864
SM	0.7822	0.5958	0.8200	0.2712	0.5226
DA	0.0310	0.1892	0.9116	0.2470	0.0788
VA	0.1236	0.2538	0.3622	0.3704	*0.6138*
L	0.9530	0.87	0.5562	0.7994	0.9074
FP	0.3142	0.7124	0.9112	0.3156	0.8874
DMN	0.0738	0.1132	0.2076	**0.001**	0.7928
**rTLE>Controls**					
VIS	0.0352	0.0380	0.0176	*0.6980*	0.3218
SM	0.0058	0.0780	0.1282	0.2332	0.1166
DA	0.9218	0.8046	0.3426	*0.9152*	0.2482
VA	0.0040	0.0098	0.0738	0.0048	0.0674
L	0.0018	0.0058	0.0496	0.0186	0.0532
FP	0.8446	0.8808	0.7062	0.9880	0.4364
DMN	0.0612	0.0340	0.1444	0.4750	0.1024

*SI Table 7: Comparison between TLE patients and healthy controls for EEG-fMRI correlation restricted to the connections of inside each intrinsic connectivity networks (Yeo et al., 2011). Dark green: Bonferroni threshold p<0.05/(7*5)~0.0014, Light green: Uncorrected threshold p<0.05.*

**lTLE<Controls**	**delta**	**theta**	**alpha**	**beta**	**gamma**
VIS	0.1074	0.2134	0.0396	0.1800	0.0930
SM	0.3530	0.8618	0.9896	0.3184	0.1246
DA	0.2590	0.5130	0.6648	0.2002	0.1620
VA	0.5356	0.6096	0.9556	0.3830	0.0376
L	0.9958	0.6205	0.8244	0.9776	0.9968
FP	0.9894	0.9978	0.9526	0.2578	0.6854
DMN	0.0198	0.0304	0.0070	0.0074	0.5592
**rTLE>Controls**					
VIS	0.9744	0.8996	0.9222	0.9742	0.5592
SM	0.0556	0.0068	0.0386	0.2798	0.8514
DA	0.7534	0.5934	0.7744	0.9486	0.2890
VA	0.0140	0.0156	0.1282	0.2472	0.7892
L	0.2154	0.5988	0.6714	0.2334	0.0612
FP	0.1952	0.0294	0.1266	0.8012	0.7908
DMN	0.8308	0.8956	0.8960	0.6592	0.7342

*SI Table 8: Comparison between TLE patients and healthy controls for the spatial contribution (Colclough et al., 2016) of intrinsic connectivity networks (Yeo et al., 2011) to the global EEG-fMRI correlation. Dark green: Bonferroni threshold p<0.05/(7*5)~0.0014, Light green: Uncorrected threshold p<0.05.*

#patient	Epilepsy lateralization	IED /minute	Total IED
**256Ch-3T**			
1	left	0	0
2	left	0	0
3	left	0	0
4	left	0	1
5	left	0	2
6	left	0	0
7	left	0	0
8	left	0	7
9	left	0	4
10	left	0	0
11	left	11.2	144
12	left	0	0
13	left	0	0
14	right	0	1
15	right	0	0
16	right	0	0
17	right	0.2	1
18	right	0	12
19	right	0	0
20	right	0	1
21	right	0	20
22	right	0	0
23	right	0.2	3
**64Ch-3T**			
24	left	0.05	1
25	left	0	0
26	left	0	0
27	right	0	0
28	right	0.05	1
29	right	0.14	3
30	right	2.19	46
31	right	0	0
32	right	0	0
33	right	0	0
34	right	0	0

SI Table 9: IEDs during recordings for each patient. Note that IED/minute in dataset 256Ch3T were based on the first 5 minutes analyzed. While total IEDs refers to the whole 20-minute recording in 256Ch3T, for the 64Ch3T both columns refer to the total recorded 21 minutes.

	delta	theta	alpha	beta	gamma
**Controls**	0.33	0.35	0.36	0.40	0.25
**lTLE**	0.28	0.32	0.35	0.31	0.24
**rTLE**	0.41	0.46	0.46	0.44	0.31
**P (5000 iterations)**	**delta**	**theta**	**alpha**	**beta**	**gamma**
**Controls > lTLE**	0.23	0.45	0.69	0.030	0.79
**Controls < rTLE**	**0.0024**	**0.0002**	**0.0004**	**0.0086**	0.021

SI Table 10: Spatial correlation between FC_{fMRI} and FC_{EEG} for each frequency band for each dataset when excluding IED rates > 1/minute (exclude patient #11 and patient #30).

	delta	theta	Alpha	beta	gamma
**All**	0.38	0.41	0.43	0.43	0.36
**Controls**	0.35	0.36	0.38	0.40	0.28
**lTLE**	0.29	0.34	0.35	0.33	0.29
**rTLE**	0.43	0.47	0.49	0.46	0.40
**P (5000 iterations)**	**delta**	**theta**	**alpha**	**beta**	**gamma**
**Controls > lTLE**	0.10	0.44	0.57	0.0516	0.89
**Controls < rTLE**	**0.002**	**0.0002**	**0.0008**	**0.0046**	**0.0006**

SI Table 11: Spatial correlation between FC_{fMRI} and FC_{EEG} for each frequency band for each dataset for z-scored connectomes (z-score normalization was carried out for each individual connectivoty matrix with respect to all connections in the connectivity matrix). Spatial correlation between FC_{fMRI} and FC_{EEG} for each frequency band averaged for each group across both datasets (5000 permutations of group labels, significant results marked in dark green bold: Bonferroni threshold p<0.05/5=0.01), Light green: Uncorrected threshold p<0.05.

	fMRI	delta	theta	alpha	beta	gamma
**Controls**	-0.39	-0.58	-0.61	-0.68	-0.67	-0.46
**lTLE**	-0.34	-0.58	-0.61	-0.64	-0.59	-0.54
**rTLE**	-0.50	-0.60	-0.65	-0.69	-0.63	-0.45
**P (5000 iterations)**		**delta**	**theta**	**alpha**	**beta**	**gamma**
**Controls > lTLE**	0.0446	0.81	0.76	0.51	0.0062	0.9974
**Controls < rTLE**	0.0088	0.085	0.045	0.10	0.83	0.27

SI Table 12: Spatial correlation between FC_{fMRI}/FC_{EEG} and Euclidian distance averaged for each group across both datasets (we tested absolute spatial correlation to match the direction of the main analysis, 5000 permutations of group labels, significant results marked in dark green bold: Bonferroni threshold p<0.05/5=0.01 Light green: Uncorrected threshold p<0.05.

	**beta**	**beta**
**Controls & lTLE**	Correlation DMN	Spatial Contribution DMN
Age	0.1738	0.0490
Sex	0.1752	0.4110
Controls vs. lTLE	***0.0016***	***0.0250***
256Ch-3T vs. 64Ch-3T	0.2098	0.4640
**lTLE & rTLE**		
Age	0.3946	0.1804
Sex	0.0704	0.0228
HS vs. Non-HS	0.0022	0.0336
Epilepsy Duration	0.3394	0.0960
IED/Min	0.2640	0.1020
lTLE vs. rTLE	***0.0010***	***0.0008***
256Ch-3T vs. 64Ch-3T	0.0082	0.0016

SI Table 13: Spatial correlation and spatial contribution between FC_{fMRI} and FC_{EEG} for each frequency band for each dataset restricted to the connections of the DMN network. P-Values of permutation test with 5000 iterations of linear model coefficients when bootstrapping averages of FC_{EEG}-FC_{fMRI} correlation in the DMN. The contrast of interest was controls vs. lTLE (dark green Bold: p<0.05) and lTLE vs. rTLE (dark green Bold: p<0.05 of contrast of interest). The Permutation test is carried out by switching labels of interest. Significant values are marked in BOLD italics. HS: hippocampal sclerosis

	delta	theta	alpha	beta	gamma
**HS**					
**Controls**	0.33	0.35	0.36	0.40	0.25
**lTLE**	0.22	0.23	0.24	0.23	0.17
**rTLE**	0.37	0.38	0.39	0.38	0.26
**P (5000 iterations)**	**delta**	**theta**	**alpha**	**beta**	**gamma**
**Controls > lTLE**	0.089	0.047	0.13	0.0022	0.53
**Controls < rTLE**	0.017	0.018	0.016	0.12	0.052
**No HS**					
**Controls**	0.33	0.35	0.36	0.40	0.25
**lTLE**	0.29	0.35	0.37	0.34	0.24
**rTLE**	0.36	0.42	0.41	0.43	0.26
**P (5000 iterations)**	**delta**	**theta**	**alpha**	**beta**	**gamma**
**Controls > lTLE**	0.69	0.92	0.96	0.63	0.93
**Controls < rTLE**	0.018	0.0008	0.0134	0.004	0.047

SI Table 14: Impact of HS on spatial correlation between FC_{fMRI} and FC_{EEG} for each frequency band averaged for each group across both datasets (5000 permutations of group labels, significant results marked in dark green bold: Bonferroni threshold p<0.05/10=0.05, Light green: Uncorrected threshold p<0.05. HS: hippocampal sclerosis).

	delta	theta	alpha	beta	gamma
**Intra-hemispheric left**					
**Controls**	0.32	0.36	0.37	0.43	0.24
**lTLE**	0.29	0.33	0.32	0.31	0.28
**rTLE**	0.41	0.46	0.49	0.47	0.37
**P (5000 iterations)**	**delta**	**theta**	**alpha**	**beta**	**gamma**
**Controls > lTLE**	0.45	0.42	0.33	0.020	0.94
**Controls < rTLE**	0.0086	**0.0044**	**0.003**	0.050	**0.0004**
**Intra-hemispheric right**					
**Controls**	0.34	0.36	0.36	0.41	0.31
**lTLE**	0.32	0.34	0.39	0.34	0.31
**rTLE**	0.43	0.46	0.47	0.47	0.36
**P (5000 iterations)**	**delta**	**theta**	**alpha**	**beta**	**gamma**
**Controls > lTLE**	0.53	0.61	0.82	0.098	0.82
**Controls < rTLE**	0.0078	**0.0024**	0.0094	0.014	0.083

SI Table 15: Spatial correlation between FC_{fMRI} and FC_{EEG} for each frequency band averaged for each group across both datasets and limited to left or right intrahemispheric connections (5000 permutations of group labels, significant results marked in dark green bold: Bonferroni threshold p<0.05/10=0.005, Light green: Uncorrected threshold p<0.05).

	delta	theta	alpha	beta	gamma
Mean correlation					
**Controls**	0.1317	0.1296	0.1257	0.1364	0.0658
**lTLE**	0.1233	0.1337	0.1263	0.1215	0.0629
**rTLE**	0.1439	0.1509	0.1442	0.1628	0.1029
**P (onesided t-test)**	**delta**	**theta**	**alpha**	**beta**	**gamma**
**Controls > lTLE**	0.30	0.60	0.51	0.19	0.44
**Controls < rTLE**	0.21	0.11	0.16	0.060	0.025

SI Table 16: Spatial correlation between FC_{fMRI} and FC_{EEG} for each frequency. Mean correlation of each frequency/group and onsided t-test across all individuals (band in dark green bold: Bonferroni threshold p<0.05/5=0.01, Light green: Uncorrected threshold p<0.05).

	delta	theta	alpha	beta	gamma
**Controls > lTLE**					
**Baseline Difference**	0.03	0.02	0.01	0.08	-0.01
**Permuted difference**	0.024 +- 0.029	0.024 +- 0.033	0.030 +- 0.039	0.026 +- 0.030	0.041 +- 0.042
**Controls < rTLE**					
**Baseline Difference**	0.09	0.11	0.12	0.05	0.07
**Permuted difference**	-0.023 +- 0.038	-0.023 +- 0.036	-0.028 +- 0.044	-0.024 +- 0.031	-0.034 +- 0.048

SI Table 17: Baseline FC_{fMRI}-FC_{EEG} spatial correlation difference between groups (for each of the two respective contrasts: r(Controls)-r(lTLE) and r(rTLE)-r(Controls)) The permuted difference depicts the mean + standard deviation of FC_{fMRI}-FC_{EEG} correlation difference distribution between random groups when permuting group labels (averaged over 5000 iterations)
